# Use of echinocandin outpatient parenteral antimicrobial therapy for the treatment of infection caused by *Candida* spp.: utilization, outcomes and impact of a change to weekly dosing

**DOI:** 10.1093/jac/dkae302

**Published:** 2024-09-11

**Authors:** Fiona Clarke, Adelaide Grenfell, Sarah Chao, Helen Richards, Tony Korman, Benjamin Rogers

**Affiliations:** Monash Infectious Diseases, Monash Health, Clayton, Victoria, Australia; Monash Doctors, Monash Health, Clayton, Victoria, Australia; Pharmacy Department, Monash Health, Clayton, Victoria, Australia; Hospital in the Home, Monash Health, Clayton, Victoria, Australia; Monash Infectious Diseases, Monash Health, Clayton, Victoria, Australia; Department of Microbiology, Monash Health, Clayton, Victoria, Australia; Centre for Inflammatory Diseases, Monash University School of Clinical Sciences at Monash Health, Clayton, Victoria, Australia; Monash Infectious Diseases, Monash Health, Clayton, Victoria, Australia; Hospital in the Home, Monash Health, Clayton, Victoria, Australia; Centre for Inflammatory Diseases, Monash University School of Clinical Sciences at Monash Health, Clayton, Victoria, Australia

## Abstract

**Background:**

Outpatient parenteral antimicrobial therapy (OPAT) can deliver extended parenteral treatment of fungal infections in an ambulatory setting, whilst minimizing treatment burden and cost. The extended dosing interval of rezafungin may potentiate the benefits of OPAT.

**Methods:**

This retrospective cohort study includes all adult patients who received echinocandin therapy in a large OPAT programme between 2012 and 2022. Patient characteristics, treatment and outcomes were studied. Data were analysed to determine the effects of replacing daily dosing with weekly dosing of echinocandin.

**Results:**

Across the study period, 11% (44/386) of all patients in our Health Service treated with ≥7 days of echinocandin were managed via OPAT. All were *Candida* and related ‘yeast-like’ species infections. *Nakaseomyces glabrata* (20/41; 49%) was the most common pathogen, fungaemia the most common presentation (17/41; 41%) and azole resistance the most frequent indication for echinocandin use (21/41; 51%).

In total, 633 days of echinocandin were administered as OPAT. Thirteen patients (13/41; 32%) received concurrent parenteral antibacterials. Treatment success was achieved in 30/41 (73%) patients. If daily echinocandin dosing was replaced with weekly dosing, a potential 52% (633 to 326) reduction in the total number of treatments (for any therapy) delivered by the OPAT team is possible. The ongoing need for daily antibacterial administration mitigated the benefit in some of this cohort.

**Conclusions:**

Echinocandin therapy can be safely delivered via OPAT with outcomes equivalent to bed-based care. The extended dosing interval of rezafungin will allow for a substantial reduction in the number of treatments required across the patient cohort.

## Introduction

Invasive fungal infections with *Candida* spp. have a high morbidity and mortality, and are increasingly common.^1,2^ In some circumstances, prolonged parenteral therapy may be required due to the infection severity, complexity and/or azole resistance.

Outpatient parenteral antimicrobial therapy (OPAT) can improve the patient experience and decrease the burden and cost of care. Antifungal therapy has been employed successfully in OPAT for stable patients, although reports of echinocandin use are limited.^3,4^ The recent introduction to the market of rezafungin, an echinocandin with once-weekly dosing,^5^ presents an opportunity to further simplify the OPAT delivery of echinocandin therapy.^6^

We aim to analyse our current use of echinocandin therapy on OPAT (using existing daily therapy) and model the feasibility and potential utility of changing to once-weekly echinocandin dosing.

## Methods

We undertook a retrospective cohort analysis over 10 years (2012–23) at Monash Health (Victoria, Australia). It is a large multi-campus Health Service, with a catchment of approximately 1.3 million residents. The OPAT programme operates within a larger medically led Hospital in the Home (HITH) programme.^6,7^ Patients are referred to the OPAT programme for antifungal therapy with a plan specified by the infectious disease team. A nurse visits the patient daily at home to administer therapy. The patient also attends a weekly onsite clinical review with an infectious disease specialist.

**Figure 1.**
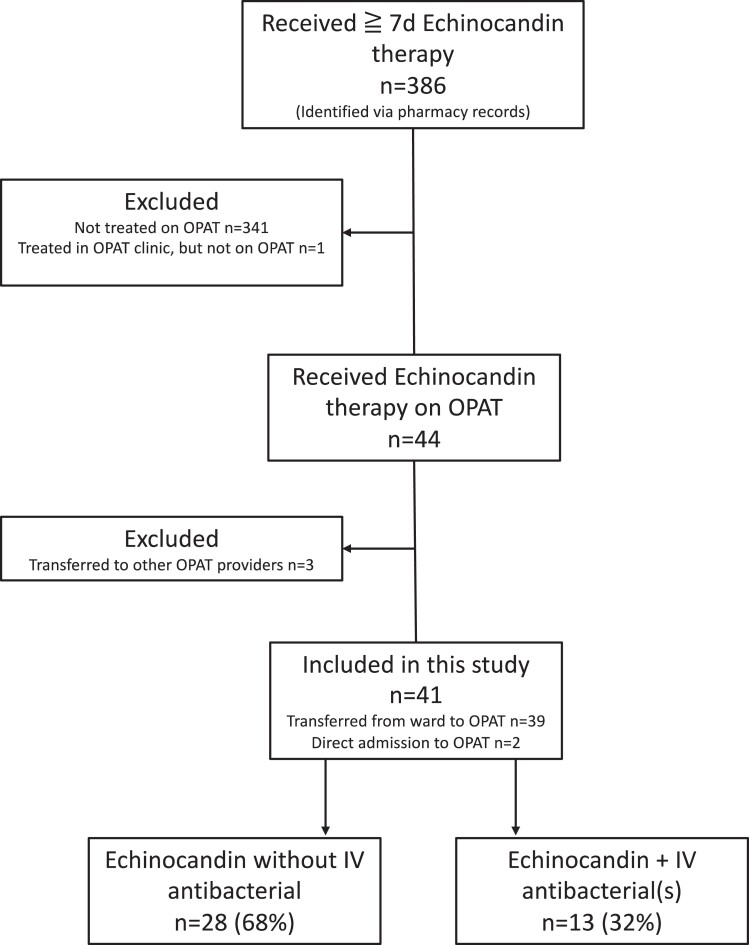
Selection of patients for study.

All adult patients (>18 years) who received echinocandin therapy on the OPAT programme were included in this study. Patients who were transferred to external OPAT providers/programmes for their care were excluded (Figure 1).

Patients treated with an echinocandin were identified through pharmacy dispensing records. Clinical data were extracted from electronic medical records and scanned paper records by two independent investigators and stored in a secure database. This project is reported as recommended by the STROBE statement.^8^

Full definitions are outlined in the Supplementary material (available as Supplementary data at *JAC* Online). In short, infection was categorized as per EORTC/MSG 2019 criteria^9^ and outcomes were adapted from the UK OPAT Good Practice Recommendations (GPR).^10^ Antifungal susceptibility testing was performed using either the VITEK 2 (bioMérieux, Marcy-l’étoile, France) or the Sensititre YeastOne (Thermo Fisher, Waltham, MA, USA).

### Data analysis

Days of echinocandin/antibacterial were calculated from medication chart records and are presented as a median and IQR. The primary analysis, to determine the feasibility of weekly echinocandin therapy, was conducted based on a potential change in the number of OPAT ‘treatments’ required if weekly rezafungin was substituted for daily echinocandin. A ‘treatment’ was defined as the administration of one or more parenteral therapies (echinocandin and/or antibacterial), where a nursing visit was required.

The replacement was calculated based on a 7 day dosing interval for rezafungin^5^ with commencement of rezafungin on the first day of OPAT care. Where the duration of echinocandin on OPAT was not a multiple of 7 days, the duration was conservatively rounded upwards to the nearest 7 days, assuming the real-life duration was the minimal acceptable (e.g. 21 days = three doses on Days 1, 8 and 15; 11 days = two doses on Days 1 and 8). To understand the impact of rounding, the excess days of therapy were calculated in this scenario based on an assumed 7 day therapeutic effect of rezafungin.

### Ethics

This project was approved as a Quality Improvement Activity by Monash Health Human Research Ethics Committee (Reference number QA/87034/MonH-2022-325894).

## Results

Over the study period, 386 patients received extended echinocandin therapy (for ≥7 days) within our health service. In total, 11% (44/386) received a proportion of their therapy via OPAT. Utilization increased over time, with the highest number of echinocandin patients on OPAT in the final year of the study, 2022 (9/41; 22%). Clinical and demographic details of the cohort are shown in Table 1. Surgery (within 30 days) and antibiotic use during the acute admission were the most frequent risk factors for fungal infection, with 25/41 (61%) of patients having at least one of these risk factors (Table S1).

**Table 1.** Description of clinical characteristics, infection microbiology and outcomes of those receiving OPAT echinocandin therapy

Characteristic	
Clinical characteristics	
Age, years, median (range)	60 (21–93)
Sex, *n* (%)	
Female	16 (39)
Male	25 (61)
Place of residence, *n* (%)	
Home	39 (95)
Residential aged care	2 (5)
Charlson comorbidity index, median (IQR)	2 (0–3)
Diagnosis requiring antifungal therapy, *n* (%)	
Fungaemia^a^	17 (41)
Intra-abdominal collection	11 (27)
Bone and joint infection	6 (15)
Other invasive candidiasis^b^	4 (10)
Oesophageal candidiasis (non-HIV related)	3 (7)
Culture results, *n* (%)	
Culture positive	37 (90)
No culture, empirical therapy	2 (5)
Histological diagnosis	2 (5)
Microbiological and treatment characteristics, *n* (%)	
Fungal species	
*Nakaseomyces glabrata (Candida glabrata)*	20 (49)
* Candida albicans*	14 (34)
* Candida parapsilosis*	4 (10)
* Pichia kudriavzevii* (*Candida krusei*)	4 (10)
* Candida tropicalis*	1 (2)
* Wickerhamomyces anomalus (Candida pelliculosa*)	1 (2)
OPAT and antifungal therapy characteristics	
Agent, *n* (%)	
Micafungin	37 (90)
Anidulafungin	3 (7)
Caspofungin	1 (2)
Indication for echinocandin therapy, *n* (%)	
Isolate not susceptible to azoles	21 (51)
Contraindication to azole therapy^c^	8 (20)
Clinical judgement^d^	12 (29)
Venous access for delivery of OPAT echinocandin, *n* (%)	
PICC	39 (95)
Peripheral IVC	2 (4)
Treatment with concurrent IV antibiotics on OPAT, *n* (%)	13 (32)
Daily administration	12 (29)
Twice-daily administration	1 (2)
Duration of admission (days), median (IQR)	
Acute admission (pre-OPAT)	17 (10–26)
OPAT	17 (8–26)
Duration of echinocandin therapy (days)	Median (IQR)
Total (acute + OPAT)	23 (14–41)
Acute	13 (7–23)
OPAT	9 (4–15)
Outcome of treatment, *n* (%)	
Successful	30 (73)
Discharged to community	29 (71)
Planned readmission	1 (2)
Unsuccessful	9 (22)
Readmitted; related to infection and/or therapy	7 (17)
Patient led cessation of therapy	2 (5)
Indeterminate	
Readmission unrelated to OPAT episode	2 (5)

IVC, intravenous catheter.

^a^Urological source, *n* = 6; line-related, *n* = 5; intra-abdominal infection, *n* = 2; unclear source, *n* = 2; endocarditis, *n* = 1; related to injecting drug use, *n* = 1.

^b^Culture sites were (1 each of) epicardial space, breast implant material, epidural abscess, pleural empyema.

^c^Drug–drug interactions, *n* = 4; liver dysfunction, *n* = 3; previous adverse effects with azoles, *n* = 1.

^d^See Table S2 for detailed description.

All patients had infections with *Candida* or related species (Table 1). Invasive fungal infection was proven (EORTC/MSG criteria) in 37/41 (90%) of patients. One patient was treated for presumed invasive infection but did not meet the criteria, and 3/41 (7%) patients had oesophageal candidiasis.

Over the 10 year study period, a total of 633 days of echinocandins were administered via OPAT. Most courses of echinocandin on OPAT were of an extended duration, with 76% more than 7 days and 46% more than 14 days. (Figure S1) Micafungin was the most commonly administered agent, as this is the default option in our hospital formulary (37/41; 90%) (Table 1).

Excluding the two patients admitted directly to the OPAT programme, echinocandin was administered for a median of 9 days (IQR 4–15) via OPAT comprising 58% of patients’ total echinocandin duration. Overall, OPAT/HITH accounted for 49% of patients’ total length of care within our Health Service (median OPAT/HITH stay of 17 days; IQR 8–26) as patients remained on the programme for care, including ongoing antibacterial and/or wound management after completion of antifungals.

Concurrent parenteral antibacterials were administered in 13/41 (32%) patients. Polymicrobial intra-abdominal collections (54%; 7/13) and collections at another site (46%; 5/13) were the most common indication. Most patients receiving concurrent parenteral antibacterials (77%; 10/13) received echinocandins via OPAT for >14 days.

### Weekly dosing of echinocandin therapy

Our 633 daily OPAT ‘treatments’ with an echinocandin equate to 106 potential treatments with a weekly echinocandin, a nominal reduction of 527/633 (92%) treatments. As 13 patients received concurrent daily parenteral antibiotics, 262 visits/administration for this therapy are still required, even with weekly echinocandin administration (42 treatments with echinocandin + antibiotic AND 220 treatments on the remaining 6 days of the week with antibiotics alone). Therefore, by utilizing weekly echinocandin in our cohort, the total care burden could be reduced from 633 to 326 treatments (52% reduction) (Figure S2).

Using the conservative weekly dosing strategy, there was a median excess therapy of 2 days (IQR 1–4) per patient, with 109 excess days across the cohort. This represents a 15% increase in the total ‘days’ of echinocandin therapy exposure relative to daily dosing.

## Discussion

Our study demonstrates OPAT echinocandin treatment as a safe and feasible alternative to inpatient administration. To our knowledge, there are no other studies of OPAT echinocandin use for *Candida* and related yeast-like species infections. Substitution with weekly echinocandin dosing may benefit the outpatient setting, reducing the total number of visits by 52%.

The use of OPAT echinocandin therapy was substantial and increased over the study period. This may be due to the convergence of the rising incidence of fungal infections^11^ and azole-non-susceptible isolates,^12^ but also increases in overall Health Service activity^13^ and a greater emphasis on the use of OPAT within healthcare over the study period.

Azole resistance was the leading indication for use of echinocandins on OPAT. This aligns with the rising rate of azole resistance amongst *Candida* spp. infections.^12,14^ Therapy was complete and uncomplicated in 73% of patients, demonstrating the overall safety of OPAT. The readmission rate (9/41; 24%) was higher than our previously described rate of 12% across the HITH programme (infectious and non-infectious diagnoses)^7^ but similar to the failure rate in clinical trials of echinocandin therapy.^15,16^ This comparatively high readmission rate suggests some caution is required in delivering care with once-weekly OPAT drug administration. The patient may require review/clinical contact with the OPAT programme more frequently than weekly to ensure adequate monitoring and early detection of failure.

This analysis of echinocandin use illustrates the potential utility of weekly echinocandin therapy in OPAT. The ReSTORE Trial demonstrated that rezafungin is non-inferior to caspofungin in candidaemia and invasive candidiasis.^16^ Substituting daily OPAT echinocandin with weekly rezafungin could benefit providers by decreasing labour associated with administration. Furthermore, patient benefits include decreased treatment burden, given weekly visits and the potential to receive extended therapy without requiring a peripherally inserted central catheter. Our findings also illustrate the importance of accounting for concurrent therapies when considering this benefit.

Our study has limitations. The generalizability of findings is limited, given data are from a single metropolitan health service in Australia. The cohort size is limited by low frequency of echinocandin use in OPAT. Retrospective review of records has limitation and subjectivity. The analysis of substitution with weekly therapy did not involve a formal economic evaluation (the agent is not currently available/priced in Australia). Finally, the infecting isolates in this study were not tested for susceptibility to rezafungin. Susceptibility was assumed based on susceptibility to other echinocandins.

## Conclusions

Echinocandins are used in the outpatient setting for a broad array of conditions, especially in the setting of antifungal resistance or when azole therapy is contraindicated. Our study is novel in demonstrating successful OPAT treatment of *Candida* and closely related yeast-like species infections with echinocandin therapy. There may be a saving of labour, decreased treatment burden for the patient and other potential benefits with weekly echinocandin dosing on OPAT. This therapy will likely offer the most benefit in patients who do not require coadministration of other therapies. A formal economic evaluation could further inform this analysis. Additionally, when ongoing clinical trials are completed, rezafungin may also emerge as an OPAT-delivered therapy for long-term antifungal prophylaxis and potentially other infections.^17,18^

## Supplementary Material

dkae302_Supplementary_Data

## Supplementary data

Figures S1 and S2 and Tables S1 and S2 are available as Supplementary data at *JAC* Online.
