# Supplementary material for: Use of echinocandin outpatient parenteral antimicrobial therapy for the treatment of infection caused by Candida spp.: utilization, outcomes and impact of a change to weekly dosing
Source: J Antimicrob Chemother. 2024 Sep 11;79(11):2896–900. doi: 10.1093/jac/dkae302 (PMC11531808; doi:10.1093/jac/dkae302)
Supplement: dkae302_Supplementary_Data [file dkae302_supplementary_data.docx]

**Supplementary Material**

DEFINITIONS

Site of infection was defined as documented by the infectious disease team. Infection was categorised as per the EORTC/MSG 2019 criteria^1^ and risk factors for invasive fungal infections as described previously. ^2, 3^

Recent was determined as within 30 days. Immunosuppression was subcategorised into medication-related, malignancy, or other immunosuppressing condition.

Indication for echinocandin therapy was categorised into; non-susceptibility to azole antifungals; contra-indication to azoles; or clinical judgement. Clinical judgement included infection or syndrome where national guidelines^4^ and/or the treating infectious diseases physician recommended echinocandin therapy OR there was clinical failure of azole therapy.

Outcomes of treatment on the OPAT program were categorised based on the UK OPAT Good Practice Recommendations (GPR)^5^ with modifications to reflect our practice. We defined ‘Successful treatment’ to include GPR definitions of ‘treatment aim attained’ but also included patients who had planned readmission as successful, rather than ‘indeterminate’. An ‘Unsuccessful’ outcome comprised ‘treatment aim not attained’ definition of patients who fail to complete OPAT and/or required unplanned re-admission.

Extended in-patient therapy was defined as 7 days or more of echinocandin therapy. Seven days was selected as a feasible time in which a patient could initiate therapy and transition to the OPAT program.

Supplementary Table 1: Description of risk factors for fungal infection in patients receiving echinocandin on OPAT

| Risk Factors for invasive fungal infection  Antibiotic use during acute admission  Surgery in last 30d  Solid tumour cancer  Diabetes mellitus  Chemotherapy, current or within the last 12 months  Central venous catheter (within last 30 days)  Prior systemic antifungal use  Other immunosuppressing condition  Trauma  Haematological malignancy  Current renal dialysis  Corticosteroids within the last year  Other immunosuppressing agents  Injecting drug use (current)  Corticosteroids (current) | 13 (32)  12 (29)  11 (27)  7 (17)  7 (17)  5 (12)  5 (12)  5 (12)  4 (10)  4 (10)  3 (7)  3 (7)  3 (7)  3 (7)  1 (2) |
| --- | --- |

Supplementary Table 2: Description of “Clinical Judgement” Indications for Echinocandin Therapy

| Peritoneal dialysis peritonitis |
| --- |
| Breast tissue expander infection |
| Empirical therapy for suspected fungal epidural abscess |
| Complex abdominal wound |
| Intra-abdominal abscess |
| Adductor canal abscess and osteomyelitis |
| Infected ureteric stent |
| Discitis and vertebral osteomyelitis |
| Infective endocarditis |
| Candidaemia (non-line related) |
| Hepatosplenic candidiasis |
| Clinical failure of oral fluconazole despite proven susceptibility |

Supplementary Figure 1: Duration of echinocandin therapy in OPAT for individual patients

Supplementary Figure 2: Comparison of number of episodes of treatment required in the current state (daily echinocandin administration) and potential replacement with weekly echinocandin administration.

References (Supplementary Material)

1. Donnelly JP, Chen SC, Kauffman CA et al. Revision and Update of the Consensus Definitions of Invasive Fungal Disease From the European Organization for Research and Treatment of Cancer and the Mycoses Study Group Education and Research Consortium. *Clinical Infectious Diseases* 2019; **71**: 1367-76.

2. Pappas PG, Kauffman CA, Andes DR et al. Clinical Practice Guideline for the Management of Candidiasis: 2016 Update by the Infectious Diseases Society of America. *Clin Infect Dis* 2016; **62**: e1-50.

3. Rüping MJ, Vehreschild JJ, Cornely OA. Patients at high risk of invasive fungal infections: when and how to treat. *Drugs* 2008; **68**: 1941-62.

4. Therapeutic Guidelines. https://www.tg.org.au.

5. Chapman ALN, Patel S, Horner C et al. Updated good practice recommendations for outpatient parenteral antimicrobial therapy (OPAT) in adults and children in the UK. *JAC Antimicrob Resist* 2019; **1**: dlz026.
